# An Atypical Presentation of Pulmonary Oedema Secondary to Suspected Anabolic Steroid-Induced Cardiomyopathy

**DOI:** 10.7759/cureus.101364

**Published:** 2026-01-12

**Authors:** Ami Davies

**Affiliations:** 1 General Medicine, Eastbourne District General Hospital, Eastbourne, GBR

**Keywords:** acute cardiogenic pulmonary edema, anabolic-androgenic steroid, heart failure with reduced ejection fraction, steroid-induced cardiomyopathy, transthoracic echocardiogram

## Abstract

A case report of a patient presenting with acute shortness of breath and chest pain, on a background of anabolic-androgenic steroid (AAS) use. An initial chest radiograph and a CT scan showed bilateral pulmonary infiltrates suggestive of atypical infection or lung malignancy. However, further evaluation confirmed these findings to be due to cardiogenic pulmonary oedema resulting from left ventricular failure, secondary to suspected anabolic steroid-induced cardiomyopathy. The patient’s symptoms improved following the initiation of guideline-directed medical therapy for heart failure, in conjunction with oral diuretics. Further MRI imaging confirmed a significantly hypertrophied left ventricle with marked systolic dysfunction. Whilst excluding an ischaemic cardiomyopathy as the cause, it did also suggest the presence of a bystander infarct. This case highlights the spectrum of cardiovascular disease that can develop with prolonged use of anabolic steroids and underscores the heterogeneous radiological presentations of acute pulmonary oedema.

## Introduction

In England and Wales, there has been a gradual increase in the use of anabolic-androgenic steroids (AAS), peaking at 411,000 adults in 2016-2017 [1]. Global AAS use is substantial and has continued to rise over the past two decades [2]. A 2014 meta-analysis reported an overall prevalence of 3.3% in the global population, with the highest rates observed in the Middle East and South America [3]. In the United States, it is estimated that more than three million individuals engage in anabolic steroid use [4].

Common side effects associated with AAS use include oligoazospermia, alopecia, and depression [5]. However, a growing body of literature has documented the adverse cardiovascular consequences associated with supratherapeutic AAS use, including cardiomyopathy, myocardial infarction, arrhythmias, and venous thromboembolism [6-9]. Proposed pathophysiological mechanisms include the development of myocardial hypertrophy and the induction of a hypercoagulable state. Additionally, coronary vasospasm resulting from suppressed nitric oxide production has been suggested to contribute to the underlying process [8]. This report presents a case of non-ischaemic cardiomyopathy related to AAS use, with a particular emphasis on the atypical radiological changes observed. Although cases have been reported in which cardiogenic pulmonary oedema radiologically mimics conditions such as infection or malignancy [10-12], the literature remains limited regarding the association between AAS use, left ventricular failure, and atypical imaging findings.

## Case presentation

A male in his 50s presented to the Emergency Department with a one-week history of a dry cough and worsening shortness of breath. The cough was mainly nocturnal with associated orthopnoea and paroxysmal nocturnal dyspnoea. He also reported a two-day history of left-sided chest pain that was non-radiating and worse on inspiration. He denied any infectious symptoms, syncope, haemoptysis, or weight loss. He was a non-smoker and abstained from alcohol but reported the use of anabolic steroids, including injectable testosterone and growth hormone. The first documented use occurred six years prior; however, the actual duration of use remains uncertain.

This gentleman was markedly dyspnoeic at rest and struggled to speak in full sentences. Examination revealed no wheeze, with breath sounds transmitted equally bilaterally. There was an absence of peripheral limb oedema. Observations showed an oxygen requirement of 2 L/minute to maintain saturations of 94%. Respiratory rate was 24, with a heart rate of 99 beats per minute, temperature of 36.6 degrees, and blood pressure of 161/75. ECG showed a suspected new left bundle branch block with signs of left ventricular hypertrophy. His initial troponin T (TNT) level was elevated at 77 ng/L (with a normal reference range of <14 ng/L). He was treated as a non-ST-elevation myocardial infarction (NSTEMI) on presentation and admitted as an inpatient for further investigation and management.

Initial laboratory investigations demonstrated chronic kidney disease (stage 3b) with an estimated glomerular filtration rate (eGFR) of 40 mL/min/1.73 m², along with elevated urea and creatinine levels; renal function was consistent with the patient’s baseline. B-type natriuretic peptide (BNP) and D-dimer levels were elevated, and the repeat troponin level after three hours was 74 ng/L. Other blood parameters were unremarkable: sodium, potassium, C-reactive protein (CRP), white cell count (WCC), and neutrophil levels were within normal ranges, while the lymphocyte count was mildly reduced (Table 1).

**Table 1:** Initial blood test results on arrival to the Emergency Department.

Test	Result	Normal reference range
Sodium (Na)	135	135-145 mmol/L
Potassium (K)	4.7	3.5-5.0 mmol/L
Urea	8.4	2.5-7.8 mmol/L
Creatinine	162	60-110 µmol/L
Estimated glomerular filtration rate (eGFR)	40	>90 mL/min/1.73 m²
Troponin T (TNT)	77	<14 ng/L
Troponin T (TNT) after 3 hours	74	<14 ng/L
B-type natriuretic peptide (BNP)	3723	<100 pg/mL
D-Dimer	797	<500 ng/mL FEU
C-reactive protein (CRP)	5	<5 mg/L
White cell count (WCC)	7.56	4.0-11.0 x 10^9^/L
Neutrophils	5.6	2.0-7.5 x 10^9^/L
Lymphocytes	1.11	1.5-4.0 x 10^9^/L

**Figure 1:**
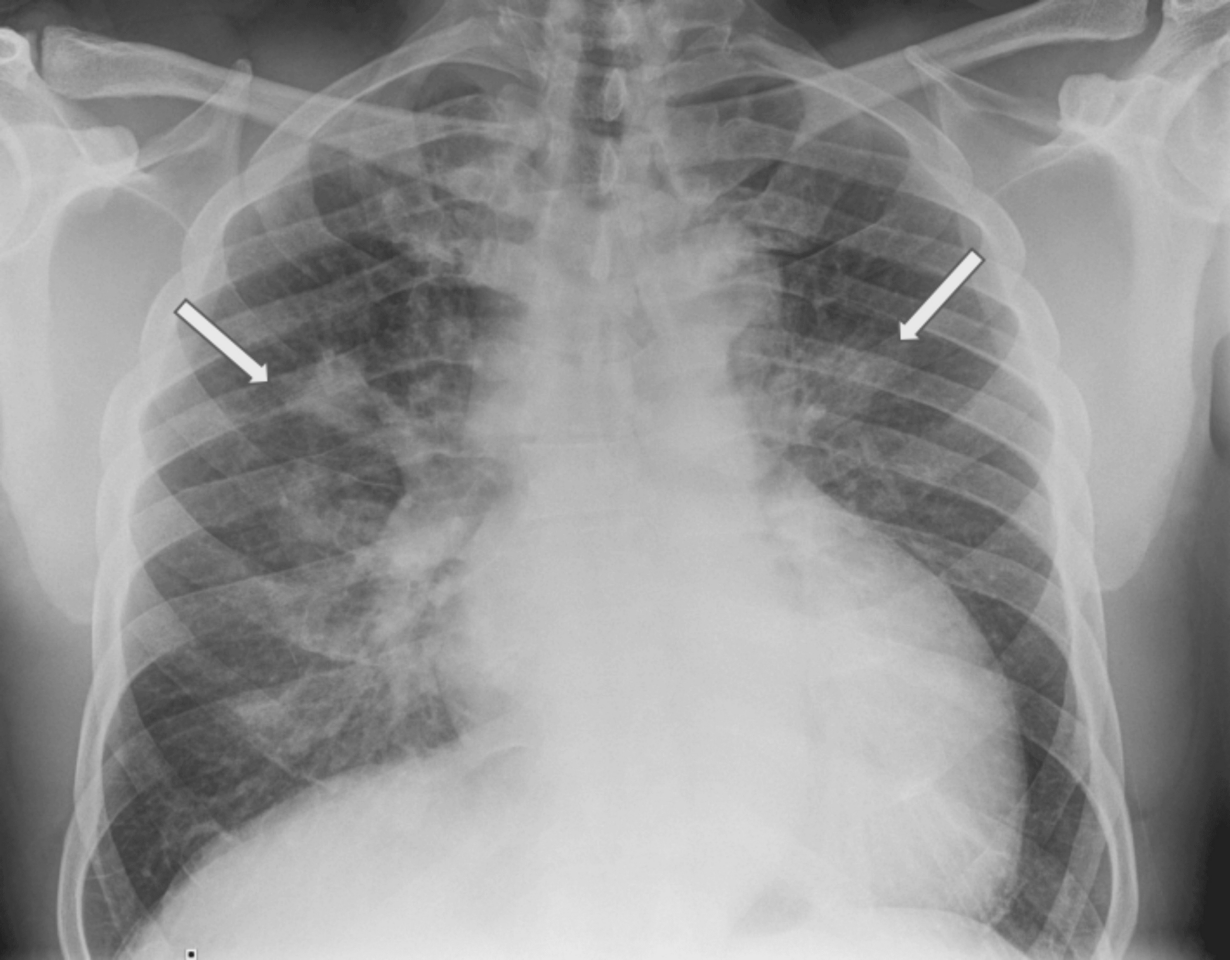
PA chest radiograph showing perihilar focal airspace shadowing (arrows) and increased cardiothoracic ratio (cardiomegaly).

**Figure 2:**
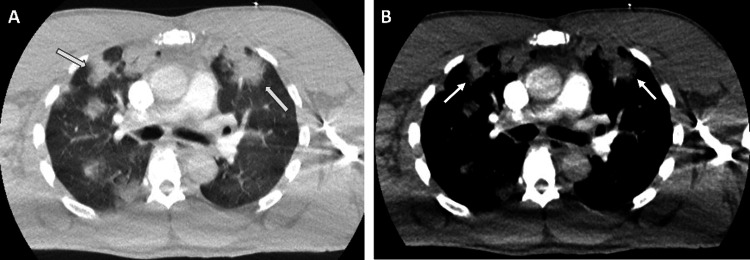
Axial CT chest images of (A) the lung parenchyma window and (B) the mediastinal window showing patchy areas of consolidation scattered throughout both lungs (arrows), predominantly in a central/perihilar distribution.

**Figure 3:**
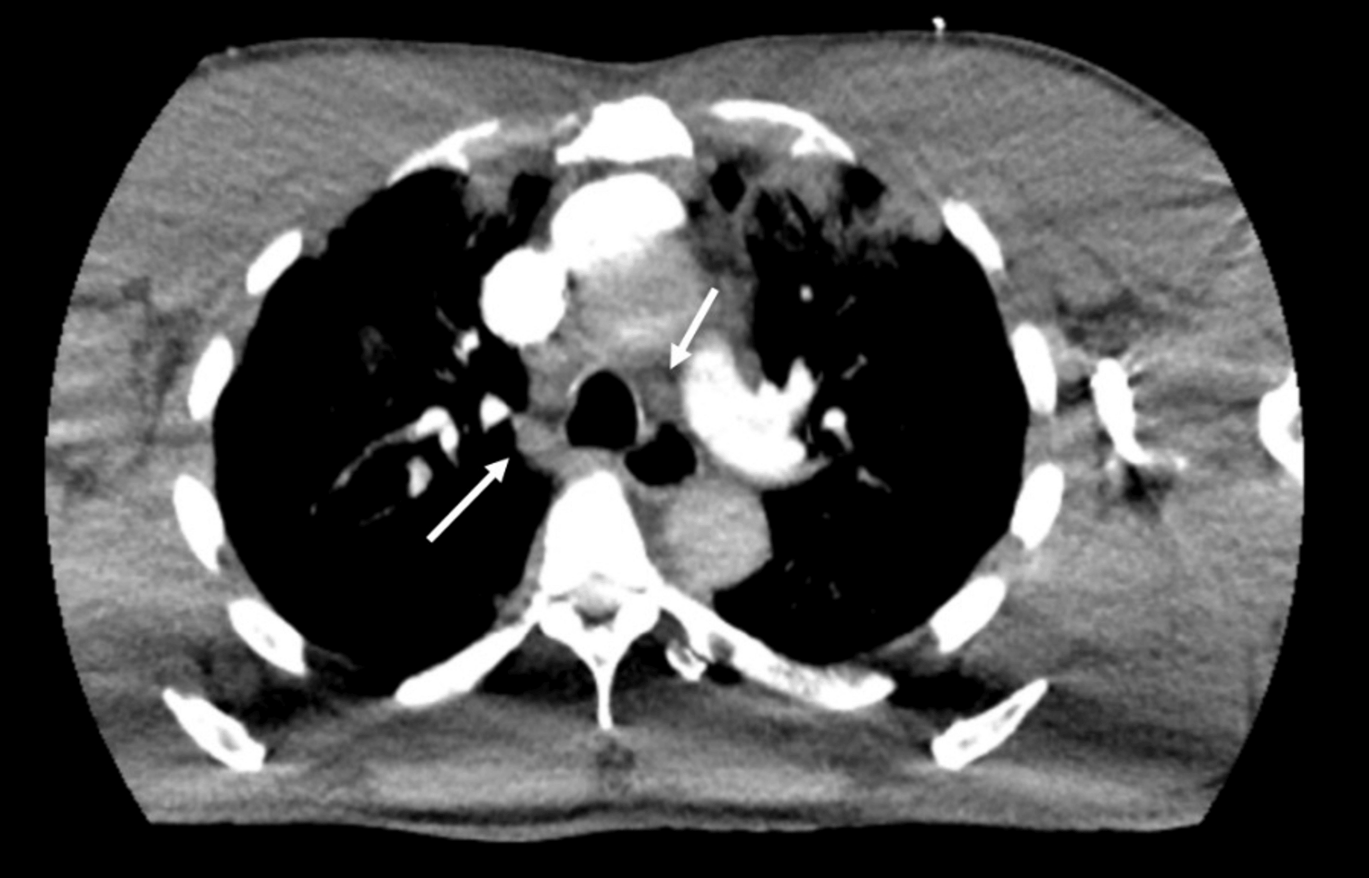
Axial CT chest images of the mediastinal window, showing bilateral lower paratracheal lymph nodes (arrows).

**Figure 4:**
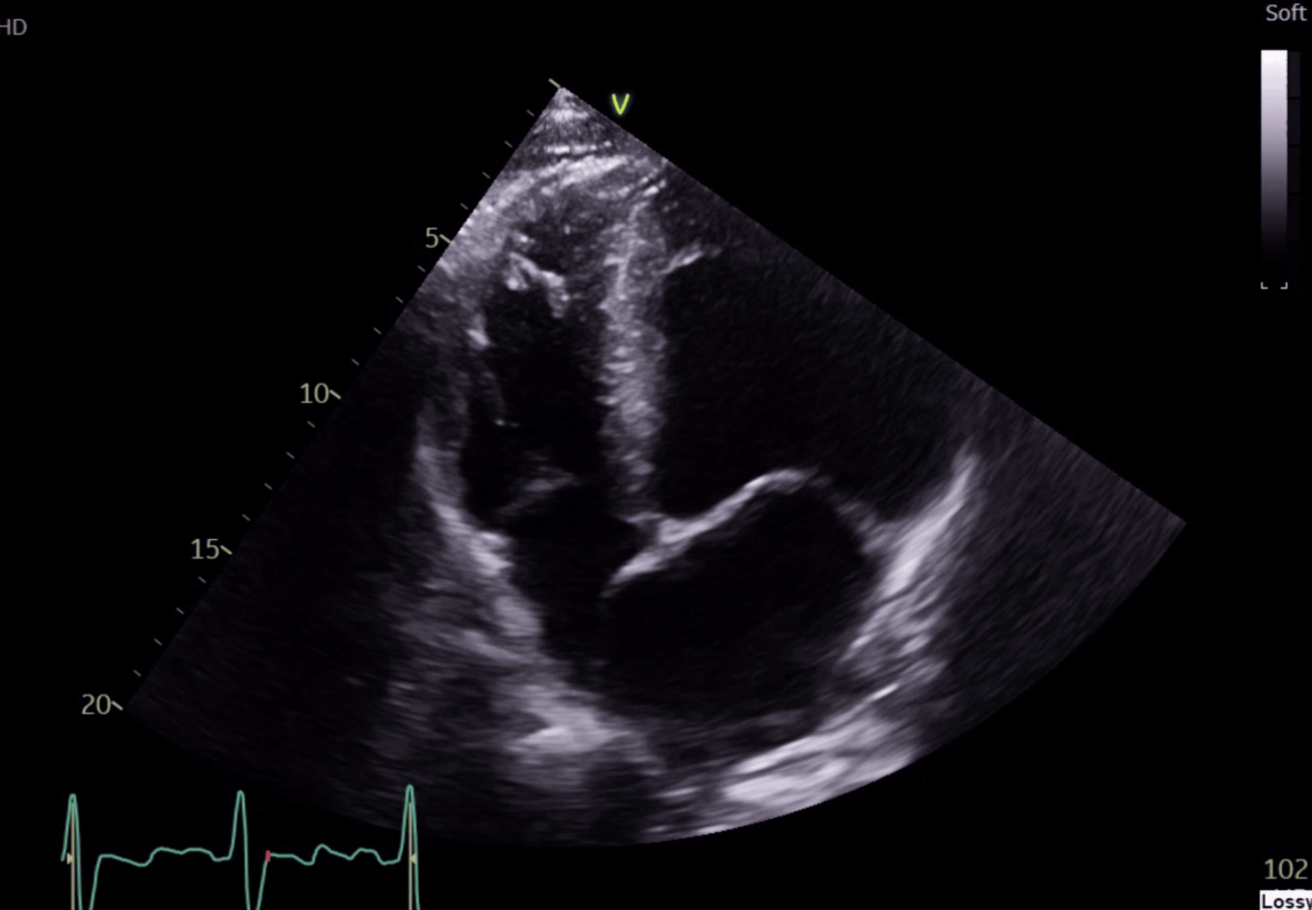
An apical four-chamber (A4C) view of the trans-thoracic echocardiogram, showing a dilated left ventricle cavity.

A chest radiograph on admission (Figure 1) showed bilateral consolidative changes in the right perihilar and lower lung regions, and an enlarged heart size. The remaining lung fields and pleural spaces were clear. A CT pulmonary angiogram demonstrated no large filling defects in the main, left, right, and proximal segmental branches of the pulmonary arteries, but it showed scattered hyperdense lesions predominantly in the upper lobes of both lungs (Figure 2), with prominent bilateral lower paratracheal lymph nodes (Figure 3). Hepatic reflux of contrast was noted. The differentials at this stage were of atypical chest infection or lung malignancy, but his atypical pneumonia screen was inconclusive for beta-D-glucan antigens and negative for *Legionella* and *Streptococcal pneumoniae* urinary antigens. The concluding impression was of pulmonary oedema. A bedside echocardiogram (Figure 4) showed a dilated left ventricular cavity and eccentric left ventricular hypertrophy (left ventricular mass of 440 g, relative wall thickness of 0.28). There was globally severely impaired left ventricular systolic function, with a visually estimated ejection fraction of 15-20%. There was dilatation of the left atrium, with mild mitral and tricuspid regurgitation, but no aortic stenosis or regurgitation.

The patient was initially treated with aspirin 300 mg, prasugrel 60 mg, and fondaparinux sodium 2.5 mg for five days. The fondaparinux sodium was subsequently discontinued once the diagnosis of a NSTEMI was excluded with serial troponin levels. A single dose of 40 mg intravenous furosemide was given in the Emergency Department, and regular furosemide 40 mg twice daily was commenced following cardiology review. Antibiotics were not commenced as the overall impression was felt to be more in keeping with pulmonary oedema as opposed to infection, given the normal inflammatory markers and clinical history. The patient was commenced on dapagliflozin 10 mg once a day and sacubitril/valsartan 24 mg/26 mg one tablet twice a day for optimisation of his heart failure management. His bisoprolol fumarate was increased from 2.5 mg once a day to twice a day, and his angiotensin-converting enzyme (ACE) inhibitor was discontinued, given his renal function.

**Figure 5:**
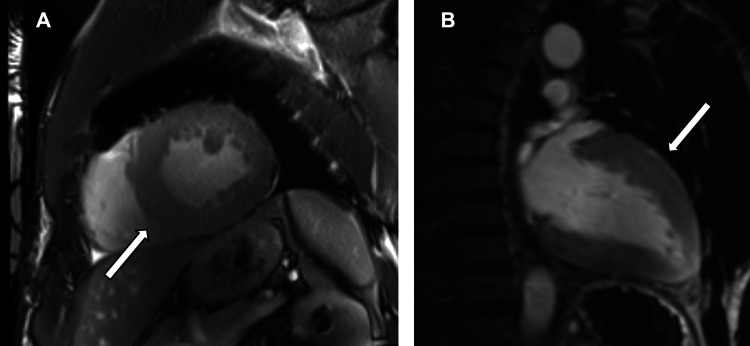
MRI cardiovascular images showing marked concentric left ventricle hypertrophy (arrows) in the (A) two-chamber (2CH) and (B) three-chamber (3CH) planes.

**Figure 6:**
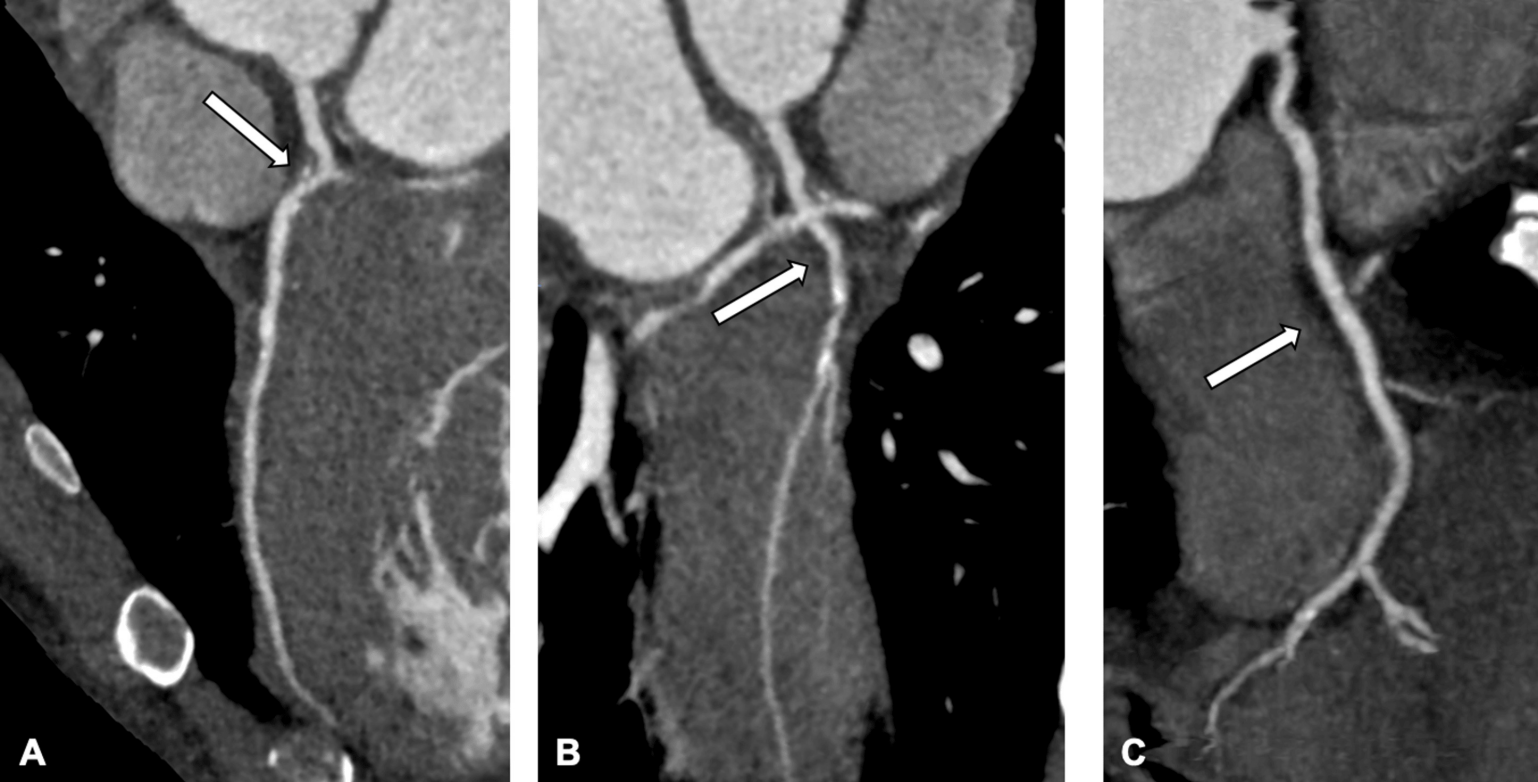
Curved multiplanar reformation images from the CTCA showing (A) mixed morphology plaque stenosis of the left anterior descending artery (B) non-calcified stenosis of the left circumflex artery after its origin and (C) diffuse disease throughout the right coronary artery.

The patient was discharged following improvement in his symptoms. Successful diuresis resulted in a 5 kg reduction in body weight from admission to discharge. He underwent an outpatient cardiac MRI, with ongoing follow-up by cardiology and the heart failure team. At his three-month review, his New York Heart Association (NYHA) Functional Classification improved from Class IV to Class I. The cardiac MRI showed global hypokinesia. There was significant left ventricular dysfunction, with significant hypertrophy of the skeletal muscle of the left ventricle (concentric hypertrophy) (Figure 5). There was dilatation of the right ventricle with a reduced ejection fraction. The findings were suggestive of a non-ischaemic cardiomyopathy; however, a focal subendocardial-to-mid wall scar in the anterolateral wall suggested the presence of an old infarct. A CT coronary angiogram (CTCA) was therefore requested, which showed diffuse three-vessel coronary artery disease (Figure 6). He is due to undergo a catheter coronary angiogram.

## Discussion

Testosterone exerts both androgenic and anabolic effects. Its androgenic function plays a key role in the development of the male reproductive system and secondary sexual characteristics, which underpins its use in individuals with hypogonadism. The anabolic function primarily promotes protein synthesis in myocytes, facilitating increased muscle mass and strength [13]. AAS are synthetic derivatives of testosterone with markedly increased anabolic activity. Supratherapeutic doses of AAS can therefore lead to significant hypertrophy of both skeletal and cardiac muscle. The development of cardiac failure in individuals using high doses of AAS is primarily due to left ventricular dysfunction. Hypertrophic changes can impair both the contractility and relaxation of the left ventricle, resulting in systolic and diastolic dysfunction [14,15].

The presence of suspected previous ischaemia in this case could additionally be the result of AAS use. AAS can increase the risk of myocardial infarction, through promoting a prothrombotic state - facilitating platelet aggregation - and additionally by causing coronary vasospasm. The oxidation of excess low-density lipoprotein-cholesterol (LDLC) produced as a result of AAS inhibits nitric oxide production, hindering coronary artery relaxation [6]. Further cardiovascular effects of significant AAS use include myocardial necrosis, where the influx of calcium into cardiomyocytes results in cell apoptosis and ventricular arrhythmias [6,7].

Despite the clinical presentation of this case suggesting congestive cardiac failure as the cause of this patient’s symptoms, the radiological appearances were suggestive of alternative diagnoses. Pulmonary oedema typically presents radiologically with ground glass opacities, bronchovascular bundle thickening, and interlobular septal thickening [16]. In cardiogenic pulmonary oedema, most often caused by left ventricular failure, bi-basal pleural effusions are a frequent additional finding. Increased hydrostatic pressure in the pulmonary vasculature, secondary to congestion, can lead to the extravasation of fluid from intravascular to extravascular spaces, resulting in widespread interstitial thickening. Therefore, the presence of numerous well-circumscribed hyperdense lesions in this patient’s CT scan presents an unusual pattern for pulmonary oedema. Differential diagnoses for the presence of scattered pulmonary opacities include infection, sarcoidosis, or malignancy. However, this patient’s history and blood results were not supportive of these diagnoses.

Non-cardiogenic causes of pulmonary oedema can also present with bilateral opacities [17]. Pulmonary haemorrhage, a known complication of supratherapeutic AAS use, can similarly present with bilateral patchy infiltrates. Nonetheless, the absence of haemoptysis and a normal haemoglobin level excluded this diagnosis. Extensive cardiac imaging ultimately demonstrated that this patient’s symptoms were due to severe left ventricular failure. Repeat imaging of the lungs following successful diuresis may have been beneficial in confirming a cardiac cause of his radiological findings.

## Conclusions

AAS use is associated with impaired ventricular function and an increased risk of acute coronary events. Despite the rising prevalence of AAS use, its cardiovascular consequences are often underrecognised by clinicians. In particular, there is a growing need to consider AAS-induced cardiomyopathy as a potential underlying cause in young patients presenting with acute cardiac dysfunction. In this case, the patient presented with acute left ventricular failure, leading to cardiogenic pulmonary oedema characterized by well-circumscribed opacities on imaging, findings attributable to significantly elevated hydrostatic pressures. Repeat imaging following diuresis can be valuable in assessing treatment response and in differentiating cardiac from non-cardiac causes of the patient’s symptoms.
